# Impact of mediastinal lymph node enlargement on the prognosis of idiopathic pulmonary fibrosis

**DOI:** 10.1371/journal.pone.0201154

**Published:** 2018-07-25

**Authors:** Sooim Sin, Kyung Hee Lee, Jee Hye Hur, Sang-hoon Lee, Yeon Joo Lee, Young-jae Cho, Ho Il Yoon, Jae Ho Lee, Choon Taek Lee, Jong Sun Park

**Affiliations:** 1 Division of Pulmonary and Critical Care Medicine, Department of Internal Medicine, Seoul National University Hospital, Seoul, Republic of Korea; 2 Department of Radiology, Seoul National University Bundang Hospital, Seongnam-Si, Gyeonggi-Do, Republic of Korea; 3 Department of Radiology, Hanil General Hospital, Seoul, Republic of Korea; 4 Division of Pulmonary and Critical Care Medicine, Department of Internal Medicine, Seoul National University College of Medicine, Seoul National University Bundang Hospital, Seongnam-Si, Gyeonggi-Do, Republic of Korea; University of Alabama at Birmingham, UNITED STATES

## Abstract

**Background:**

Mediastinal lymph node enlargement (LNE) is common in idiopathic pulmonary fibrosis (IPF) and is known to be associated with the severity of lung fibrosis. However, the relationship between mediastinal LNE and the prognosis of IPF has not been determined to date.

**Methods:**

This study included patients with IPF from the interstitial lung disease registry at Seoul National University Bundang Hospital, from January 2012 to March 2016. Two thoracic radiologists independently reviewed mediastinal LNE and lung parenchymal fibrosis and ground glass opacities in chest computed tomography scans of each patient, which were obtained upon diagnosis. Mortality and admission rates were analyzed.

**Results:**

In total, 132 patients (104 [78.8%] male; median age, 72 years; range, 51–84 years) were enrolled and 73 (55.3%) patients had mediastinal LNE (short axis ≥ 10 mm in diameter). Mortality was significantly higher among patients with LNE than among those without LNE (hazard ratio 2.26 [95% confidence interval 1.20–4.23], p = 0.011). Of the patients with LNE, 24.7% experienced acute exacerbation and 43.8% experienced hospital admission for respiratory causes, in comparison with 16.9% and 40.0% of patients without LNE respectively. Although patients with LNE had a tendency to have increased rate of acute exacerbation, it was not statistically significant.

**Conclusion:**

Mediastinal LNE in IPF is associated with increased mortality and its occurrence may be considered a poor prognostic factor in patients with IPF.

## Introduction

Idiopathic pulmonary fibrosis (IPF) is a devastating and currently incurable disease with short median survival of 3–4 years. The currently available therapies for IPF, pirfenidone and nintedanib, have limited efficacy [1–3].

IPF has several clinical phenotypes, which are classified as follows in accordance with disease progression: slowly, rapidly, and mixed progressive IPF [4, 5]. Most patients have slowly progressive IPF, of which the forced vital capacity (FVC) is reported to decline from 0.13 L to 0.21 L within a year [4]. Rapidly progressive IPF has a shorter survival than the other phenotypes, with a rapidly progressive course. Some patients with IPF (5–20%) experience acute exacerbations, which lead to a poor prognosis [6].

As the clinical course of IPF is heterogeneous, it is essential to predict the disease course and mortality early on in order to establish a therapeutic strategy and provide information to the patients. Unfortunately, at present, the methods for predicting the prognosis of IPF are limited. Various factors, including individual factors and clinical prediction models that combine individual factors, have been proposed to be useful for predicting prognosis of patients with IPF [4, 5, 7–10], but these were found to lack accuracy and have not been fully validated. Even the Gender–Age-Physiology (GAP) Index [11], which is the most convenient and commonly used model for predicting mortality in IPF, has limitations, as it is not able to assess the underlying pathobiology directly [12]. Difficulty in predicting disease prognosis is a major obstacle for determining appropriate treatment timing, predicting therapeutic response, and determining the treatment group, leading to despair among clinicians and patients with IPF.

In contrast, lymph node enlargement (LNE) in IPF is a common finding, and is reported in 40–60% of patients [13–16]. The reason for mediastinal LNE in patients with IPF is unknown. However, unlike the ambiguous explanation for the development of LNE caused by a hyperplastic reaction due to a chronic inflammatory process [13, 17], recent studies have shown experimental results that can correlate the development of the lymphatics with the incidence of IPF and the progression of tissue fibrosis [18–22].

Considering this association, we hypothesized that LNE in IPF might be related to the disease severity; moreover, it could be a factor predicting the prognosis of the disease. In this study, we therefore evaluated the clinical significance of mediastinal LNE and its impact on the prognosis of IPF.

## Methods

### Study subjects and design

This study retrospectively reviewed 205 patients who were enrolled in the interstitial lung disease (ILD) registry at the Seoul National University Bundang Hospital, from January 2012 to March 2016. Patients with IPF diagnosed on the basis of the American Thoracic Society/European Respiratory Society guidelines [3], who had undergone chest computed tomography (CT) within 1 year of diagnosis, were included. Patients with concurrent pulmonary infection, such as pneumonia or tuberculosis on chest CT were excluded; those with malignancy or AIDS or uncontrolled heart disease, and those undergoing chemotherapy were also excluded. Data regarding baseline lung function, hospitalization, and death were obtained from the registry data. Information on the additional deaths of patients who were lost to follow-up was supplemented from the Korea National Statistical Office. The GAP stage [11] was calculated, and treatment with pirfenidone or nintedanib was also investigated. All patients were followed up until September 2016.

This study was approved by the Institutional Review Board of the Seoul National University Bundang Hospital (IRB No. B-1606/352-102) and conformed to the tenets of the Declaration of Helsinki. All data used in this study were fully anonymized before we access and written informed consent was obtained from all subjects.

### Chest CT evaluation

Chest CTs of each patient, obtained within 1 year from diagnosis, were reviewed independently by two thoracic radiologists at Seoul National University Bundang Hospital. When readings about LNE were discrepant between the two radiologists, readings were reconsidered and decided by a third party at the Seoul National University Bundang Hospital. Mediastinal lymph nodes were assessed using only the soft tissue windows (level 35 HU, width 450 HY). Using Mountain–Dresler modification of the ATS map (MD-ATS) [23], 2R, 2L, 3, 4R, 4L, 5, 6, and 7 nodes were reviewed and LNE was defined as a node with a short-axis diameter of 10 mm or more. Each radiologist described the presence, number, size, and location of the LNE. Interobserver agreement between the two observers was measured using kappa statistics.

In addition, the ground glass opacity score (GGO score) and fibrosis score were calculated to assess radiological disease severity, employing methods used in previous reports; both scores were calculated in accordance with the percentage of each lung lobe invaded, and the scores for the individual lobes were averaged [14]. The mean of the final scores of the two radiologists was then determined for each patient.

### Statistical analysis

Patients were initially classified into two groups depending on the presence or absence of LNE. A *t*-test was used for between-group comparisons of demographics involving continuous variables and the chi-square test and linear-by-linear association method for comparisons involving categorical variables. We performed Kaplan–Meier analysis with log-rank testing to compare all-cause mortality according to the presence of LNE. Cox proportional hazard regression analysis was used to calculate crude and adjusted hazard ratios and their 95% confidence intervals (CIs). To evaluate the discriminative ability, we calculated the C statistics and 95% CIs of univariate and multivariate Cox models. The proportional hazard assumption was evaluated by goodness-of-fit statistics (Hosmer-Lemeshow test) for the Cox model. Negative binomial regression was used to analyze factors affecting hospitalization. STATA 12 (Stata Corp, College Station, TX, USA) was used for statistical analysis. P-values less than 0.05 were considered statistically significant.

## Result

### Baseline characteristics of patients

**Fig 1:**
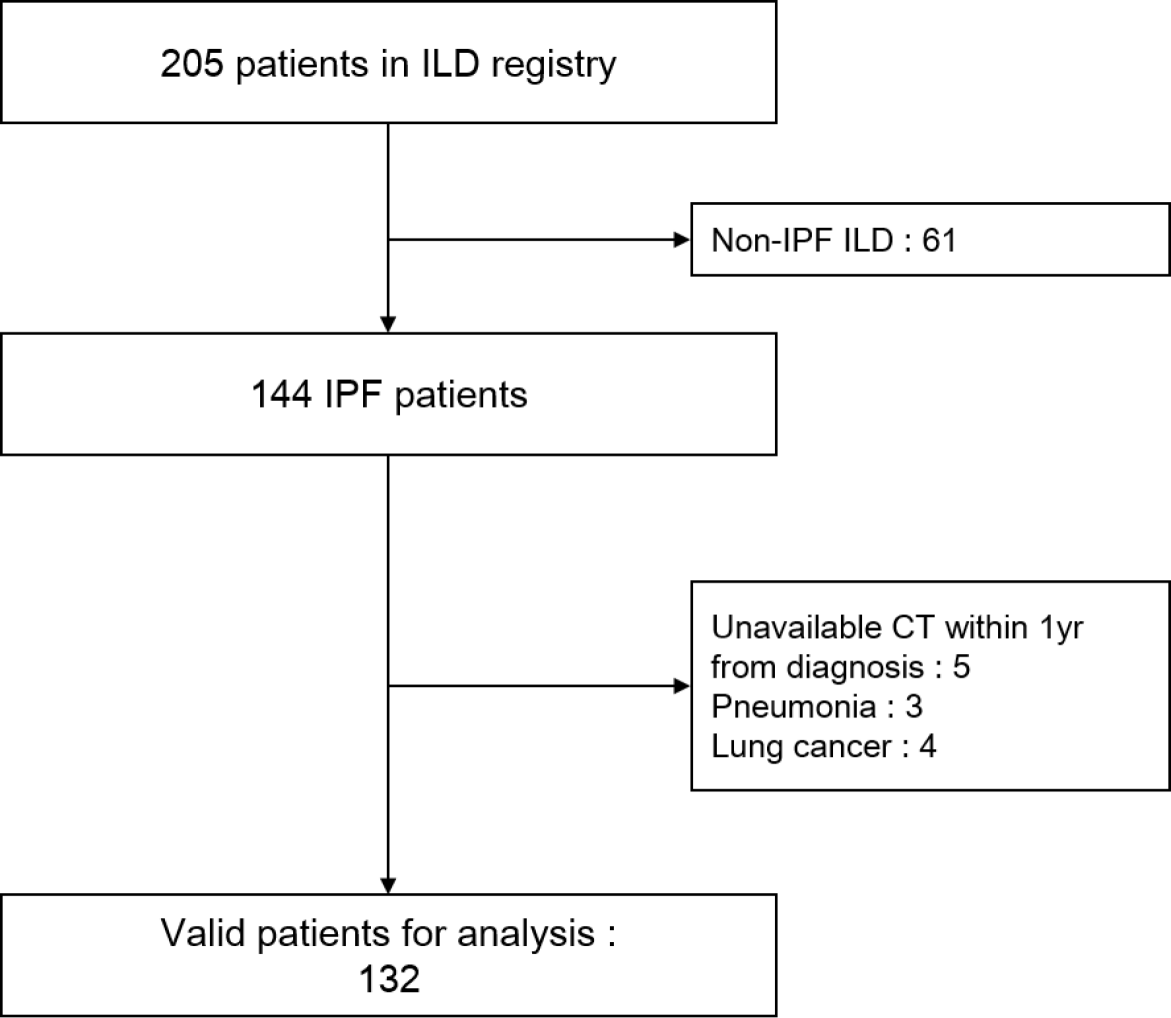
Study population from the interstitial lung disease registry. CT = computed tomography; ILD = interstitial lung disease; IPF = idiopathic pulmonary fibrosis.

Of the 205 patients included in the ILD registry, 73 patients were excluded, mainly because they were diagnosed with ILD other than IPF. Finally, 132 patients with IPF were included in the analysis (Fig 1). Demographics, GAP stage, and lung function are shown in Table 1. Of the cohort, 73 patients (55.3%) had at least one LNE in a chest CT scan that was performed within 1 year of their diagnosis, while 59 patients (44.7%) had no LNE (Table 1). Interobserver agreement between the two radiologists was substantial (kappa = 0.91). There was no statistically significant difference in age, body mass index, sex ratio, smoker proportion, or other etiologies between the groups (Table 1). Overall 14 patients (10.6%) had biopsy-proven IPF (Table 1). LNE was significantly associated with a higher GAP stage and lower diffusing capacity of the lungs for carbon monoxide (DLCO) percentage predicted (Table 1). Similarly, patients with LNE had a tendency to have lower FVC percentage and forced expiratory volume in 1 second (FEV_1_) percentage predicted. However, this difference was not statistically significant (Table 1).

**Table 1:** Demographic and clinical characteristics of patients with IPF, according to the presence of LNE.

	Overall study cohort n = 132	LNE n = 73	No LNE n = 59	p-value
**Age (y)**	72 (66–76)	72 (66–77)	71 (66–76)	0.613
**BMI (kg/m**^**2**^**)**	24 ± 2.7	24 ± 2.5	24 ± 2.8	0.823
**Male**	104 (78.8)	55 (75.3)	49 (83.1)	0.281
**Smoker**				0.725
never smoker	34 (26.2)	20 (27.8)	14 (24.1)	
ever smoker	79 (60.8)	44 (61.1)	35 (60.3)	
current smoker	17 (13.1)	8 (11.1)	9 (15.5)	
**DM**	40 (30.3)	22 (30.1)	18 (31.5)	0.963
**HTN**	48 (36.4)	24 (32.9)	24 (40.7)	0.354
**Heart disease^†^**	31 (23.5)	17 (23.3)	14 (23.7)	0.953
**GAP stage^‡^**				0.013
1	95 (72.5)	47 (65.3)	48 (81.4)	
2	29 (22.1)	18 (25.0)	11 (18.6)	
3	7 (5.3)	7 (9.7)	0 (0)	
**PFT**				
FVC, % predicted	84.4 ± 17.2	82.3 ± 16.5	86.9 ± 17.8	0.131
FEV_1_, % predicted	96.0 ± 19.7	94.4 ± 19.0	97.9 ± 20.4	0.319
DLCO,% predicted	76.3 ± 21.5	70.8 ± 21.7	82.8 ± 19.5	0.002
**Biopsy specimen proven**	14 (10.6)	9 (12.3)	5 (8.5)	0.475
**Treatment**^**§**^	31 (23.5)	16 (21.9)	15 (25.4)	0.637
**CT findings**				
Ground glass score	1.7 ± 0.6	1.9 ± 0.8	1.4 ± 0.8	< 0.001
Fibrosis score	1.6 ± 0.5	1.7 ± 0.6	1.5 ± 0.6	0.049

Median (IQR) is presented in Age, otherwise, n (%) or mean ± SD is presented for each parameter.

^†^Heart disease includes heart failure, ischemic heart disease, and valvular heart disease.

^‡^By linear-by-linear association method.

^§^Patients treated with pirfenidone or nintedanib.

BMI, body mass index; DLCO, diffusing capacity of the lungs for carbon monoxide; DM, diabetes mellitus; FVC, forced vital capacity; FEV1, forced expiratory volume in 1 second; GAP, Gender-Age-Physiology index; HTN, hypertension; IPF, idiopathic pulmonary fibrosis; LNE, lymph node enlargement; PFT, pulmonary function test.

In patients with mediastinal LNE, the total number of lymph nodes showing enlargement was 123, and 98 lymph nodes were mainly located in 7 and 4R (S1 Table). The patients with 1–2 LNEs accounted for 46.9% of the total cohort, while the patients with 3–5 LNEs accounted for 8.4% of patients (S1 Table). In a comparison of the GGO score and fibrosis score according to the presence of LNE, these scores were significantly higher among patients with than among those without LNE, suggesting that LNE is associated with disease severity (Table 1).

### Mortality and hospitalization

**Fig 2:**
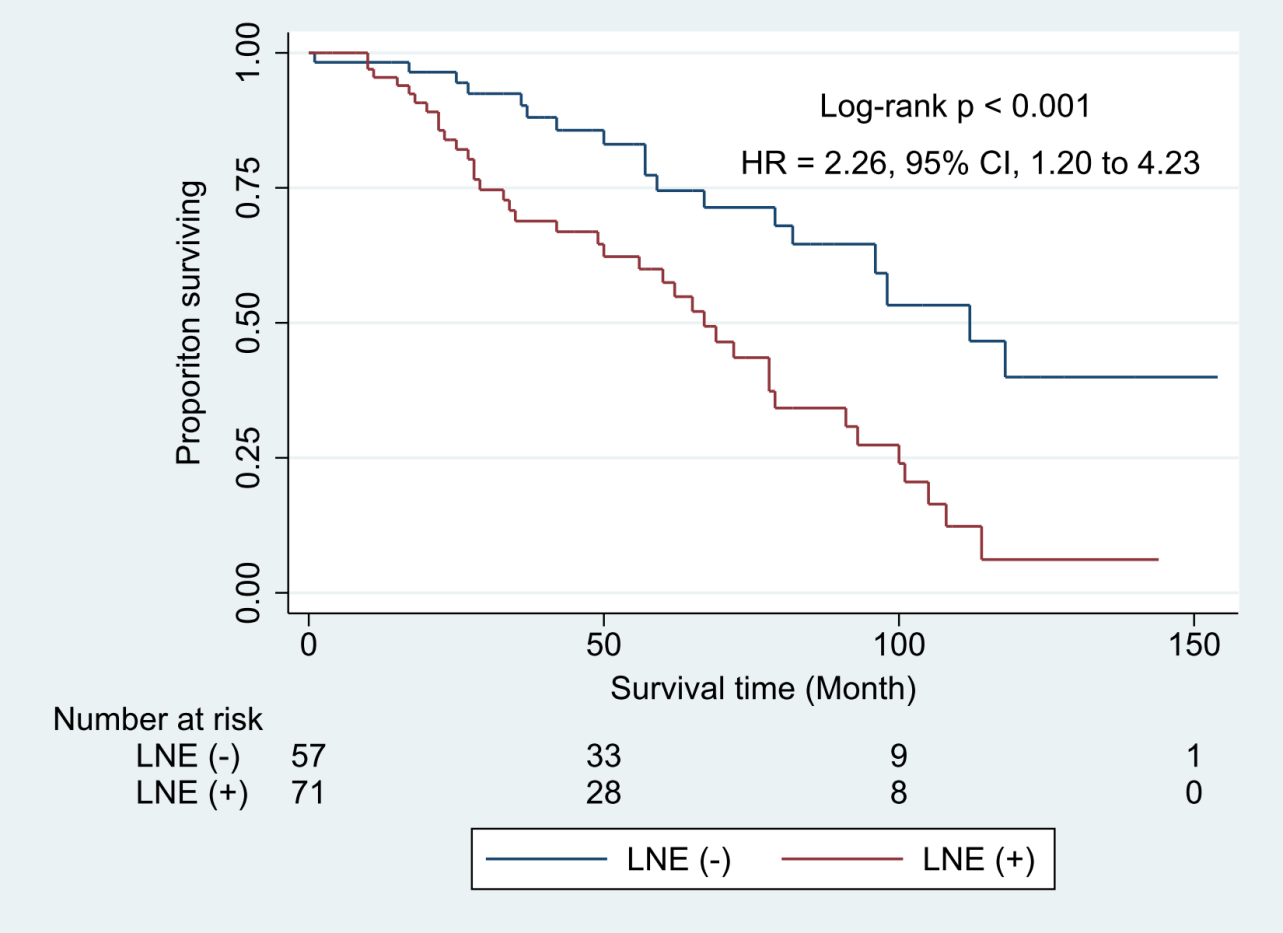
Kaplan–Meier survival curve during the follow-up period according to the presence of lymph node enlargement. p-value was obtained by log-rank test. HR and 95% CI were obtained by multivariate cox regression with model 2 of Table 3. CI = confidence interval HR = hazard ratio LNE = lymph node enlargement.

IPF patients with LNE showed a significantly lower survival rate than those without LNE (P < 0.001; Fig 2). When the crude hazard ratio was calculated through Cox proportional hazard regression, old age, lower FVC percentage predicted, and high GAP stage were significantly associated with increased mortality. The mortality rate was significantly higher (2.67-folds) in patients with than in those without LNE (p = 0.001); mortality rate increased with the increase in the number of LNEs (Table 2). The fibrosis score, but not the GGO score, was associated with mortality (Table 2).

**Table 2:** Predictors of all-cause mortality in IPF patients, according to univariate analysis.

	Univariate HR (95% CI)	p-value
**Age**	1.06 (1.02–1.10)	0.009
**Female sex**	1.06 (0.55–2.05)	0.859
**Ever-smoker vs Never smoker**	0.79 (0.43–1.43)	0.431
**PFT**		
FVC (% pred.)	0.99 (0.97–0.99)	0.044
FEV1 (% pred.)	1.00 (0.98–1.01)	0.757
DLCO (% pred.)	0.99 (0.98–1.00)	0.272
**GAP stage**		
Stage 2 vs. 1	2.04 (1.13–3.69)	0.018
Stage 3 vs. 1	3.67 (1.11–12.07)	0.032
**LNE(+) vs. LNE(-)**	2.67 (1.51–4.72)	0.001
**Number of LNE (n)**		
1–2 vs. 0	2.46 (1.36–4.46)	0.003
3–5 vs. 0	3.84 (1.65–8.90)	0.002
**Fibrosis score**	3.55 (2.02–6.24)	<0.001
**Ground glass score**	1.48 (0.98–2.23)	0.063

Univariate Cox proportional hazard regression analyses were performed to evaluate each variable’s prognostic significance. CI, confidence interval; DLCO, diffusing capacity of the lungs for carbon monoxide; FVC, forced vital capacity; FEV1, forced expiratory volume in 1 second; GAP, Gender-Age-Physiology index; HR, hazard ratio; IPF, idiopathic pulmonary fibrosis; LNE, lymph node enlargement; PFT, pulmonary function test

When multivariate analysis was performed, LNE was an independent predictor of mortality after adjusting for age, sex, FVC (% predicted), DLCO (% predicted) and fibrosis score (Table 3). Similarly, we also found that as the number of LNEs increases, the mortality rate tends to increase, after adjusting for the same variables (Table 3).

**Table 3:** Two models for identifying predictors of all-cause mortality in IPF patients by multivariate analysis.

	Model 1^†^		Model 2^‡^		Model 3^§^	
	aHR (95% CI)	p-value	aHR (95% CI)	p-value	aHR (95% CI)	p-value
**Age**	1.05 (1.01–1.10)	0.018	1.05 (1.01–1.10)	0.027	1.05 (1.01–1.10)	0.024
**Female sex**	0.92 (0.44–1.91)	0.826	1.00 (0.47–2.11)	0.996	1.04 (0.49–2.20)	0.924
**FVC (% pred.)**	0.98 (0.97–1.00)	0.092	0.98 (0.97–1.00)	0.101	0.98 (0.96–1.00)	0.084
**DLCO (% pred.)**	1.00 (0.98–1.01)	0.709	1.00 (0.99–1.02)	0.658	1.00 (0.99–1.02)	0.582
**LNE(+) vs. LNE(-)**			2.26 (1.20–4.23)	0.011		
**Number of LNE**						
1–2 vs. 0					2.14 (1.12–4.08)	0.022
3–5 vs. 0					3.16 (1.21–8.23)	0.019
**Fibrosis score**	2.63 (1.49–4.65)	0.001	2.47 (1.41–4.34)	0.002	2.39 (1.36–4.19)	0.003

Multivariate Cox proportional hazard regression analyses were performed for three survival models.

^†^Model 1 is composed of age, sex, FVC (% predicted), DLCO (% predicted), and fibrosis score.

^‡^Model 2 is composed of age, sex, FVC (% predicted), DLCO (% predicted), presence of LNE, and fibrosis score.

^§^Model 3 is composed of age, sex, FVC (% predicted), DLCO (% predicted), number of LNE, and fibrosis score.

aHR, adjusted hazard ratio; CI, confidence interval; DLCO, diffusing capacity of the lungs for carbon monoxide; FVC, forced vital capacity; IPF, idiopathic pulmonary fibrosis; LNE, lymph node enlargement.

The C statistic values for the model including the presence of LNE and number of LNE were 0.741 (95% CI, 0.661–0.821), and 0.737 (95% CI, 0.657–0.818) respectively. These values were higher than that of the model including only age, sex, FVC (% predicted), DLCO (% predicted) and fibrosis score (C statistic value, 0.708, 95% CI, 0.627–0.789).

Of the patients with LNE, 24.7% experienced acute exacerbation and 43.8% experienced hospital admission for respiratory causes, in comparison with 16.9% and 40.0% of patients without LNE respectively. Although patients with LNE tended to have an increased rate of acute exacerbation, this was not statistically significant (Table 4).

**Table 4:** Effect of LNE on hospital admission rate and acute exacerbation in IPF patients.

	Adjusted Incidence Rate Ratio (95% CI)	
	Hospitalization^†^	Acute exacerbation of IPF
**No LNE**	1 (reference)	1 (reference)
**LNE**	1.59 (0.91–2.79)	1.99 (0.89–4.49)
***p*-value**	0.102	0.093

Based on Poisson regression, multivariate analyses, adjusted for GAP stage and fibrosis score, were performed.

^†^Hospitalization for respiratory causes

CI, confidence interval; IPF, idiopathic pulmonary fibrosis, LNE, lymph node enlargement

## Discussion

Predicting the course and prognosis of patients with IPF is a very important for deciding on treatment or providing appropriate explanations to patients, this complicated by the marked heterogeneity in the clinical phenotype of IPF [4]. It has been suggested that individual factors, such as the level of dyspnea, DLCO, 6-min walking test, the extent of honeycomb on high-resolution CT, and pulmonary hypertension increase mortality [24]. In addition, composite scoring systems, such as the composite physiological index, clinical/radiological/physiological score, or GAP score, which incorporate clinical, physiological, and radiologic variables, have been proposed for predicting mortality and disease progression [7, 11, 25, 26]. Validation of these systems have also been attempted [11, 27], but the clinical phenotype and mortality of individual patients with IPF remain difficult to predict, and further studies and attempts at validations are still underway [28]. In Korea and Japan in particular, the GAP model did not predict the mortality of IPF patients accurately [29, 30]. Recently, genetic and molecular factors have been investigated for use in phenotyping and predicting mortality, but these studies are incomplete and require validation before they can be implemented clinically [8, 31–33]. Therefore, further studies are required to improve prognosis of patients with IPF.

We here first evaluated the clinical significance of mediastinal LNE in patients with IPF. Previous studies [13, 15] have reported that mediastinal LNE was associated with the fibrosis score in patients with IPF. However, no previous studies to date have evaluated the effect of LNE on the clinical outcome or prognosis in patients with IPF. Mediastinal LNE was common (55.3%) in patients with IPF and correlated with disease severity in terms of the fibrosis score. Furthermore, in this study, mediastinal LNE was an independent predictor of mortality, even after adjusting for age, sex, FVC (% predicted), DLCO (% predicted) and fibrosis score. The discriminatory ability of mortality prediction model with LNE was better than that with typically known factors.

The reason for the poor survival among patients with IPF with enlarged mediastinal LNs is unclear. LNE may represent increased lymphangiogenesis and lymphatic remodeling in IPF that may occur early after lung injury and may trigger inflammation, contributing to the progression of fibrosis [34, 35]. Currently there are several pathophysiological lines of evidence to explain this association. Several studies have investigated the frequency and location of thoracic LNE in IPF, assuming that the lymphatics are implicitly associated with fibrotic lung disease [13, 15]. In addition, studies on the severity of disease or differential diagnosis in idiopathic interstitial pneumonia associated with LNE have been performed against the same background [14, 16]. Moreover the studies about LNE with IPF indicating mediastinal LNE is a common feature in IPF [13–16], recent studies have provided more information on the role of the lymphatics in IPF by using a pathophysiological approach. El-Chemaly et al. found that lymphatic vessel area increases in parallel with disease severity and the molecular and cellular pathways associated with this abnormal lymphangiogenesis in IPF [18, 21], and a subsequent study confirmed this finding by comparing the amount of lymphatics in normal lungs and in lungs with fibrotic lung disease [20]. Moreover, the potential role of lymphatics in lung fibrosis has been suggested in mouse model [22]. In addition, lymphangiogenesis itself may be considered a therapeutic target for modulating inflammatory conditions [19]; therefore, lymphatics may be considered as a factor with a profound effect on inflammation and the accompanying fibrosis in IPF.

Transient enlargement of mediastinal LNs can also result from infection or malignancy. Therefore, we excluded patients with concomitant pneumonia, tuberculosis, uncontrolled heart disease, or active malignancy at the time of initial diagnosis. During the follow-up period, two patients who were included in analysis developed lung cancer.

Acute exacerbation is unpredictable, fatal, and has a significant impact on hospital admission in IPF. Therefore, we investigated whether LNE can predict acute exacerbation of, or hospital admission of patients with IPF. Although there was a tendency to increased rate of acute exacerbation and hospitalization in patients with LNE, the rate of acute exacerbation or hospital admission did not differ statistically significantly between patients with or without LNE.

This study has several limitations. First, although we used prospective ILD cohort, this study was analyzed retrospectively. Therefore additional prospectively designed studies are needed to determine the prognostic factor. Second, patients included in this study have relatively milder disease than other IPF cohort study patiens. Because the prospective ILD registry enrolled patients only from the outpatients clinic, patients with relatively mild disease were included, and thus the results of this study cannot be generalized to IPF patients as a whole.

In this study, mediastinal LNE was used for predicting IPF prognosis, which has not been reported previously. In addition to the known factors, LNE may serve as a new factor for more accurate prediction of mortality, providing additional information. Additionally, LNE is a very useful baseline factor that can easily be obtained through chest CT at the time of diagnosis. In particular, even after correcting for variables that are known to be associated with the prognosis of IPF, mediastinal LNE per se was an independent predictor of mortality in IPF.

In conclusion, our study revealed that mediastinal LNE could be used to predict poor prognosis of patients with IPF. We also show that the presence of LNE as well as the number of LNs showing enlargement can be used for risk stratification. Thus, LNE is likely to be a useful marker to help clinicians manage patients with IPF.

## Supporting information

S1 Table Distribution of enlarged mediastinal LNs according to location and number. (DOCX)

S1 File Raw data. (XLSX)

## Abbreviations

BMI: body mass index
CT: computed tomography
DLCO: diffusing capacity of the lungs for carbon monoxide
DM: diabetes mellitus
FEV1: forced expiratory volume in 1 second
FVC: forced vital capacity
GAP index: Gender-Age-Physiology index
GGO: ground glass opacity
HTN: hypertension
IIP: idiopathic interstitial pneumonia
ILD: interstitial lung disease
IPF: idiopathic pulmonary fibrosis
LN: lymph node
LNE: lymph node enlargement

## References

[1] King TE Jr, Bradford WZ, Castro-Bernardini S, Fagan EA, Glaspole I, Glassberg MK, et al A phase 3 trial of pirfenidone in patients with idiopathic pulmonary fibrosis. N Engl J Med. 2014;2014(370):2083–92. 10.1056/NEJMoa1402582 24836312

[2] Richeldi L, du Bois RM, Raghu G, Azuma A, Brown KK, Costabel U, et al Efficacy and safety of nintedanib in idiopathic pulmonary fibrosis. N Engl J Med. 2014;370(22):2071–82. 10.1056/NEJMoa1402584 24836310

[3] Raghu G, Collard HR, Egan JJ, Martinez FJ, Behr J, Brown KK, et al An official ATS/ERS/JRS/ALAT statement: idiopathic pulmonary fibrosis: evidence-based guidelines for diagnosis and management. Am J Respir Crit Care Med. 2011;183(6):788–824. Epub 2011/04/08. 10.1164/rccm.2009-040GL . 21471066 PMC5450933

[4] Ley B, Collard HR, King TE Jr. Clinical course and prediction of survival in idiopathic pulmonary fibrosis. Am J Respir Crit Care Med. 2011;183(4):431–40. 10.1164/rccm.201006-0894CI 20935110

[5] King TE, Pardo A, Selman M. Idiopathic pulmonary fibrosis. The Lancet. 2011;378(9807):1949–61. 10.1016/s0140-6736(11)60052-4 21719092

[6] Collard HR, Moore BB, Flaherty KR, Brown KK, Kaner RJ, King TE Jr, et al Acute exacerbations of idiopathic pulmonary fibrosis. Am J Respir Crit Care Med. 2007;176(7):636–43. 10.1164/rccm.200703-463PP 17585107 PMC2094133

[7] King TE Jr., Tooze JA, Schwarz MI, Brown KR, Cherniack RM. Predicting survival in idiopathic pulmonary fibrosis: scoring system and survival model. Am J Respir Crit Care Med. 2001;164(7):1171–81. Epub 2001/10/24. 10.1164/ajrccm.164.7.2003140 . 11673205

[8] Daccord C, Maher TM. Recent advances in understanding idiopathic pulmonary fibrosis. F1000Research. 2016;5. 10.12688/f1000research.8209.1 PMC4890320 27303645

[9] Kolb M, Collard HR. Staging of idiopathic pulmonary fibrosis: past, present and future. European Respiratory Review. 2014;23(132):220–4. 10.1183/09059180.00002114 24881076 PMC9487566

[10] Ley B, Elicker BM, Hartman TE, Ryerson CJ, Vittinghoff E, Ryu JH, et al Idiopathic pulmonary fibrosis: CT and risk of death. Radiology. 2014;273(2):570–9. Epub 2014/06/14. 10.1148/radiol.14130216 ; PubMed Central PMCID: PMCPMC4334234. 24927326 PMC4334234

[11] Ley B, Ryerson CJ, Vittinghoff E, Ryu JH, Tomassetti S, Lee JS, et al A multidimensional index and staging system for idiopathic pulmonary fibrosis. Ann Intern Med. 2012;156(10):684–91. 10.7326/0003-4819-156-10-201205150-00004 22586007

[12] Johannson KA, Ley B, Collard HR. Models of disease behavior in idiopathic pulmonary fibrosis. BMC Med. 2015;13(1):165. 26400574 10.1186/s12916-015-0403-7 PMC4581470

[13] Bergin C, Castellino RA. Mediastinal lymph node enlargement on CT scans in patients with usual interstitial pneumonitis. AJR Am J Roentgenol. 1990;154(2):251–4. Epub 1990/02/01. 10.2214/ajr.154.2.2105008 . 2105008

[14] Jung JI, Kim HH, Jung YJ, Park SH, Lee JM, Hahn ST. Mediastinal lymphadenopathy in pulmonary fibrosis: correlation with disease severity. J Comput Assist Tomogr. 2000;24(5):706–10. Epub 2000/10/25. . 11045689 10.1097/00004728-200009000-00007

[15] Attili AK, Kazerooni EA, Gross BH, Flaherty KR, Martinez FJ. Thoracic lymph node enlargement in usual interstitial pneumonitis and nonspecific-interstitial pneumonitis: prevalence, correlation with disease activity and temporal evolution. J Thorac Imaging. 2006;21(4):288–92. 10.1097/01.rti.0000213562.55914.9a 17110853

[16] Souza CA, NL, Lee KS, Johkoh T, Mitsuhiro H, Chong S. Idiopathic interstitial pneumonias: prevalence of mediastinal lymph node enlargement in 206 patients. American Journal of Roentgenology. 2006;186(4):995–9. 10.2214/AJR.04.1663 16554569

[17] Garber SJ, Wells AU, Dubois RM, Hansell DM. ENLARGED MEDIASTINAL LYMPH-NODES IN THE FIBROSING ALVEOLITIS OF SYSTEMIC-SCLEROSIS. Br J Radiol. 1992;65(779):983–6. PubMed PMID: WOS:A1992JY78000006. 10.1259/0007-1285-65-779-983 1450835

[18] El-Chemaly S, Pacheco-Rodriguez G, Ikeda Y, Malide D, Moss J. Lymphatics in Idiopathic Pulmonary Fibrosis: New Insights into an Old Disease. Lymphat Res Biol. 2009;7(4):197–203. 10.1089/lrb.2009.0014 ; PubMed Central PMCID: PMCPMC2883488. 20143918 PMC2883488

[19] Kim H, Kataru RP, Koh GY. Inflammation-associated lymphangiogenesis: a double-edged sword? The Journal of Clinical Investigation. 2014;124(3):936–42. 10.1172/JCI71607 24590279 PMC3938274

[20] Lara AR, Cosgrove GP, Janssen WJ, Huie TJ, Burnham EL, Heinz DE, et al INcreased lymphatic vessel length is associated with the fibroblast reticulum and disease severity in usual interstitial pneumonia and nonspecific interstitial pneumonia. Chest. 2012;142(6):1569–76. 10.1378/chest.12-0029 22797508 PMC3515029

[21] El-Chemaly S, Malide D, Zudaire E, Ikeda Y, Weinberg BA, Pacheco-Rodriguez G, et al Abnormal lymphangiogenesis in idiopathic pulmonary fibrosis with insights into cellular and molecular mechanisms. Proc Natl Acad Sci U S A. 2009;106(10):3958–63. Epub 2009/02/25. 10.1073/pnas.0813368106 ; PubMed Central PMCID: PMCPMC2646625. 19237567 PMC2646625

[22] Cui Y, Wilder J, Rietz C, Gigliotti A, Tang X, Shi Y, et al Radiation-induced impairment in lung lymphatic vasculature. Lymphat Res Biol. 2014;12(4):238–50. Epub 2014/11/21. 10.1089/lrb.2014.0012 ; PubMed Central PMCID: PMCPMC4267131. 25412238 PMC4267131

[23] Mountain CF, Dresler CM. Regional lymph node classification for lung cancer staging. Chest. 1997;111(6):1718–23. Epub 1997/06/01. . 9187199 10.1378/chest.111.6.1718

[24] Travis WD, Costabel U, Hansell DM, King TE Jr., Lynch DA, Nicholson AG, et al An official American Thoracic Society/European Respiratory Society statement: Update of the international multidisciplinary classification of the idiopathic interstitial pneumonias. Am J Respir Crit Care Med. 2013;188(6):733–48. 10.1164/rccm.201308-1483ST . 24032382 PMC5803655

[25] Wells AU, Desai SR, Rubens MB, Goh NS, Cramer D, Nicholson AG, et al Idiopathic pulmonary fibrosis: a composite physiologic index derived from disease extent observed by computed tomography. American journal of respiratory and critical care medicine. 2003;167(7):962–9. 10.1164/rccm.2111053 12663338

[26] Watters LC, King TE, Schwarz MI, Waldron JA, Stanford RE, Cherniack RM. A clinical, radiographic, and physiologic scoring system for the longitudinal assessment of patients with idiopathic pulmonary fibrosis. Am Rev Respir Dis. 1986;133(1):97–103. Epub 1986/01/01. 10.1164/arrd.1986.133.1.97 . 3942381

[27] Ley B, Elicker BM, Hartman TE, Ryerson CJ, Vittinghoff E, Ryu JH, et al Idiopathic pulmonary fibrosis: CT and risk of death. Radiology. 2014;273(2):570–9. 10.1148/radiol.14130216 24927326 PMC4334234

[28] Salisbury ML, Xia M, Zhou Y, Murray S, Tayob N, Brown KK, et al Idiopathic Pulmonary Fibrosis: Gender-Age-Physiology Index Stage for Predicting Future Lung Function Decline. CHEST Journal. 2016;149(2):491–8. 10.1378/chest.15-0530 PMC4944785 26425858

[29] Kim ES, Choi SM, Lee J, Park YS, Lee CH, Yim JJ, et al Validation of the GAP score in Korean patients with idiopathic pulmonary fibrosis. Chest. 2015;147(2):430–7. Epub 2014/09/12. 10.1378/chest.14-0453 . 25211374

[30] Kondoh S, Chiba H, Nishikiori H, Umeda Y, Kuronuma K, Otsuka M, et al Validation of the Japanese disease severity classification and the GAP model in Japanese patients with idiopathic pulmonary fibrosis. Respiratory Investigation. 2016;54(5):327–33. 10.1016/j.resinv.2016.02.009 27566380

[31] Pathak RR, Dave V. Integrating omics technologies to study pulmonary physiology and pathology at the systems level. Cell Physiol Biochem. 2014;33(5):1239–60. 10.1159/000358693 24802001 PMC4396816

[32] Spagnolo P, Tzouvelekis A, Maher TM. Personalized medicine in idiopathic pulmonary fibrosis: facts and promises. Curr Opin Pulm Med. 2015;21(5):470–8. 10.1097/MCP.0000000000000187 26132817

[33] Seibold MA, Wise AL, Speer MC, Steele MP, Brown KK, Loyd JE, et al A common MUC5B promoter polymorphism and pulmonary fibrosis. N Engl J Med. 2011;364(16):1503–12. 10.1056/NEJMoa1013660 ; PubMed Central PMCID: PMC3379886. 21506741 PMC3379886

[34] Mh S, L A. Lymphangiogenesis and its Role in Physiologic Wound Healing and the Pathogenesis of Pulmonary Fibrosis2015.

[35] Stump B, Cui Y, Kidambi P, Lamattina AM, El-Chemaly S. Lymphatic Changes in Respiratory Diseases: More than Just Remodeling of the Lung? Am J Respir Cell Mol Biol. 2017;57(3):272–9. Epub 2017/04/27. 10.1165/rcmb.2016-0290TR ; PubMed Central PMCID: PMCPMC5625224. 28443685 PMC5625224

