# Supplementary material for: Impact of mediastinal lymph node enlargement on the prognosis of idiopathic pulmonary fibrosis
Source: PLoS One. 2018 Jul 25;13(7):e0201154. doi: 10.1371/journal.pone.0201154 (PMC6059471; doi:10.1371/journal.pone.0201154)
Supplement: S1 Table — (DOCX) [file pone.0201154.s001.docx]

**S1 Table.** Distribution of enlarged mediastinal LNs according to location and number

| Location of LNE | Number of LN (n = 123) |
| --- | --- |
| **2L (Lt. upper paratracheal LN)** | 0 (0) |
| **2R( Rt. Upper paratracheal LN )** | 6 (4.9) |
| **4L ( Lt. lower paratracheal LN )** | 13 (10.6) |
| **4R( Rt. lower paratracheal LN )** | 35 (28.5) |
| **5 ( Subaortic LN )** | 4 (3.3) |
| **6 ( Para-aortic LN )** | 2 (1.6) |
| **7 ( Subcarinal )** | 63 (51.2) |
| **Total number of LNE** | **Number of patients (n = 132)** |
| **0** | 59 (44.7) |
| **1**−**2** | 62 (46.9) |
| **3**−**5** | 11 (8.4) |

LN : lymph node; LNE : lymph node enlargement
